# The indirect effect of peer problems on adolescent depression through nucleus accumbens volume alteration

**DOI:** 10.1038/s41598-020-69769-3

**Published:** 2020-07-30

**Authors:** Kyung Hwa Lee, Jae Hyun Yoo, Jung Lee, Seong Hae Kim, Ji Youn Han, Soon-Beom Hong, Jiyoon Shin, Soo-Churl Cho, Jae-Won Kim, David A. Brent

**Affiliations:** 1 Division of Child and Adolescent Psychiatry, Department of Psychiatry, Seoul National University Hospital, Seoul National University College of Medicine, 101 Daehak-Ro, Chongno-Gu, Seoul, 03080 Republic of Korea; 2 Department of Psychiatry, Seoul St. Mary’s Hospital, College of Medicine, The Catholic University of Korea, Seoul, Republic of Korea; 3 Integrative Care Hub, Children’s Hospital, Seoul National University Hospital, Seoul, Republic of Korea; 4 Department of Psychiatry, Korea Armed Forces Capital Hospital, Bundang, Republic of Korea; 5 Department of Psychiatry, University of Pittsburgh School of Medicine, Pittsburgh, PA USA

**Keywords:** Neuroscience, Medical research

## Abstract

Literature suggests that neurobiological factors such as brain structure play an important role in linking social stress with depression in adolescence. We aimed to examine the role of subcortical volumetric alteration in the association between peer problems as one type of social stress and adolescent depression. We hypothesized that there would be indirect effects of peer problems on adolescent depression through subcortical volumetric alteration. Seventy eight adolescents with major depressive disorder (MDD) (age mean [SD] = 14.9 ± 1.5, 56 girls) and 47 healthy controls [14.3 ± 1.4, 26 girls]) participated in this study. High-resolution structural T1 images were collected using the Siemens 3T MR scanner. Subcortical volumes were segmented using the Freesurfer 6.0 package. Peer problems were assessed using the Peer-Victimization Scale and the Bullying-Behavior Scale. There was a significant indirect effect of peer problems on adolescent depression through nucleus accumbens (NAcc) volume alteration, but not through the amygdala and hippocampal volumes. This result supported our model, which stated that peer problems have indirect effects through subcortical volumetric alteration (i.e., increased NAcc volume) on adolescent depression. Our finding suggests that altered NAcc volume may serve as a pathway, through which peer problems as one type of social stressor contribute to adolescent depression.

## Introduction

Adolescents experience peer problems (e.g., involvement in bully-victim problems) as they devote more time and effort to peer relationships^1^. The mean prevalence rates of traditional bullying and cyberbullying involvement were approximately 35% and 15% across 80 studies^2^. These peer problems such as being a bully and a victim are considered to be major social stressors, which are known to be related to important adolescent public health issues^3^. For example, youth who were bullies and victims showed higher rates of concurrent depression^3^ and reported to having long-term adverse effects with greater rates of adulthood depression and suicidality^4^. A recent study showed that the prevalence rates of both peer problems and depression remained high in 2015, or even increased compared to those in 2005^5^, indicating that the associations between peer problems and adolescent depression may continue to affect significant public health problems.

To ameliorate such public health problems associated with peer relational problems and adolescent depression, other factors should be considered. It has been suggested that neurobiological factors (e.g., brain structure and function)^6^ may play an important role in linking social stress with adolescent depression. Furthermore, adolescence is a developmental period for remodeling brain structures and increasing neural plasticity in response to social stress^7^. Brain structure may dramatically change in order to adapt to different social and emotional stresses (e.g., problematic peer relations such as bullying involvement) that frequently occur during adolescence. Brain structure alteration is also known to be associated with adolescent depression^8^.

Of particular interest, structural alteration (e.g., volumetric changes) in subcortical regions including the amygdala, hippocampus, and NAcc has been known to be associated with both social stress and psychopathology^9,10^. This may be because these regions have been implicated in social and emotion processing and reward-punishment processing^11,12^. These regions are also known to be sensitive to social stress^13^, and continue to develop throughout the adolescence^14^. It is important to investigate how peer problems, as one type of social stress, subcortical structural alteration, and depression are related in order to find potentially effective ways to help the adolescents who suffer from peer problems and depression. We proposed one possible model that may explain how these variables are related: peer problems may be indirectly associated with adolescent depression through subcortical structural alteration.

Consistent with our proposed model, animal models of depression have suggested that social stress plays critical roles in shaping subcortical brain development and induces depressive-like behaviors^15^. Previous animal studies demonstrated that social stress (e.g., social defeat) produced morphologic changes (e.g., increased or reduced spine density) in subcortical regions such as the amygdala, hippocampus, and NAcc during adolescence^16–18^. Furthermore, social stress provoked depressive-like behaviors such as increased immobility, social avoidance and helplessness in adolescent animals^16^. Previous studies using magnetic resonance imaging (MRI) and post-mortem histologic analyses showed positive associations between spine density and structural changes (e.g., volume)^19–21^. These results support the notion that altered spine density (e.g., increased spine density) may indicate volumetric alteration (e.g., increased structural volumes). Given that the brain volumetric changes were associated with cellular changes such as dendritic spine density^19–21^, previous studies suggested that social stress may contribute to subcortical structure alteration, which is thought to be associated with increased depressive-like behaviors.

There was also human adolescent research examining whether subcortical structural alteration was associated with social stress and depression. Previous adolescent studies investigating the relationship between social stress and subcortical structural alteration have been conducted in the context of family-related stress (e.g., negative mother–child interactions and emotional neglect by caregivers)^22–25^, but not in the context of peer stress (e.g., involvement in bully-victim problems). Thus, the lack of evidence in peer contexts suggests that studies are needed to improve our understanding on how peer problems are associated with subcortical structural alteration in adolescence. Previous studies on structural alteration in adolescent depression have shown that depressed youth have smaller hippocampal^26^, amygdala^27^, and NAcc volumes^8^ compared to healthy controls. However, other studies showed no differences in hippocampal and amygdala volumes between adolescents with depression or subthreshold depression and healthy controls^28,29^. Such mixed findings suggest that more research is needed to better understand relationships between subcortical volumetric alteration and adolescent depression. More importantly, there is limited evidence on the role of subcortical structural alteration in linking peer problems with adolescent depression.

In this study, we aimed to examine the indirect effects of peer problems on adolescent depression through subcortical volumetric alteration. To do this, we collected the structural images using magnetic resonance imaging (MRI) and the scores of peer problems (bullying involvement as victims and bullies) in adolescents with depression and without depression. We hypothesized that there would be significant indirect effects of peer problems on adolescent depression via volumetric alteration in subcortical regions including the amygdala, hippocampus, and NAcc. Given that previous findings were mixed and research remains relatively scarce, we did not formulate specific hypotheses on whether larger or smaller subcortical volumes would be associated with peer problems and adolescent depression.

## Results

### Demographic, clinical, and subcortical volume characteristics

Demographic, clinical, and subcortical volume characteristics are presented in Table 1.

**Table 1:** Demographic, clinical and structural characteristics.

	CON (N = 47)	MDD (N = 78)	Statistics	*p*	Effect size
Female, N (%)	26 (55.3%)	56 (71.8%)	*x*^2^ = 3.53	0.06	
Age, M (SD)	14.3 (1.4)	14.9 (1.5)	*t* = 2.23	< .05	Cohen’s *d* = 0.41
Intelligence quotient (IQ), M (SD)	109.6 (10.7)	105.3 (13.9)	*t* = 1.95	0.07	
Depressive symptoms (CDRS-R), M (SD)	22.7 (4.4)	58.8 (11.7)	*F* = 367.8^a^	< .001	Partial *η*^2^= 0.75
Anxiety symptoms (SCARED), M (SD)^c^	10.7 (10.1)	36.7 (16.2)	*F* = 98.60^a^	< .001	Partial *η*^2^= 0.45
Peer problems (PVS/BBS), M (SD)	3.2 (0.7)	4.1 (1.1)	*F* = 24.53^a^	< .001	Partial *η*^2^= 0.17
Subcortical volumes (mm^3^)					
Amygdala, M (SD)	3,298.3 (345.5)	3,270.0 (369.1)	*F* = 0.22^b^	0.64	
Hippocampus, M (SD)	8,167.0 (627.1)	8,266.2 (741.2)	*F* = 1.28^b^	0.26	
NAcc, M (SD)	878.3 (110.2)	945.0 (134.3)	*F* = 10.81^b^	< .005	Partial *η*^2^= 0.08
Intracranial volume, M (SD)	1,519,375.6 (128,157.7)	1,532,970.3 (140,467.0)	*t* = 0.54	0.59	

*CON* healthy controls, *MDD* major depressive disorder, *CDRS-R* children’s depression rating scale-revised, *SCARED* the screen for child anxiety related emotional disorders, *PVS/BBS* peer-vicitmization scale/bullying-behavior scale, *NAcc* nucleus accumbens, *M* mean, *SD* standardized deviation.

^a^Adjusted for gender, age, and IQ.

^b^Adjsuted for gender, age, IQ, and intracranial volume (ICV).

^c^One outlier in the CON group was identified and removed.

**Figure 1:**
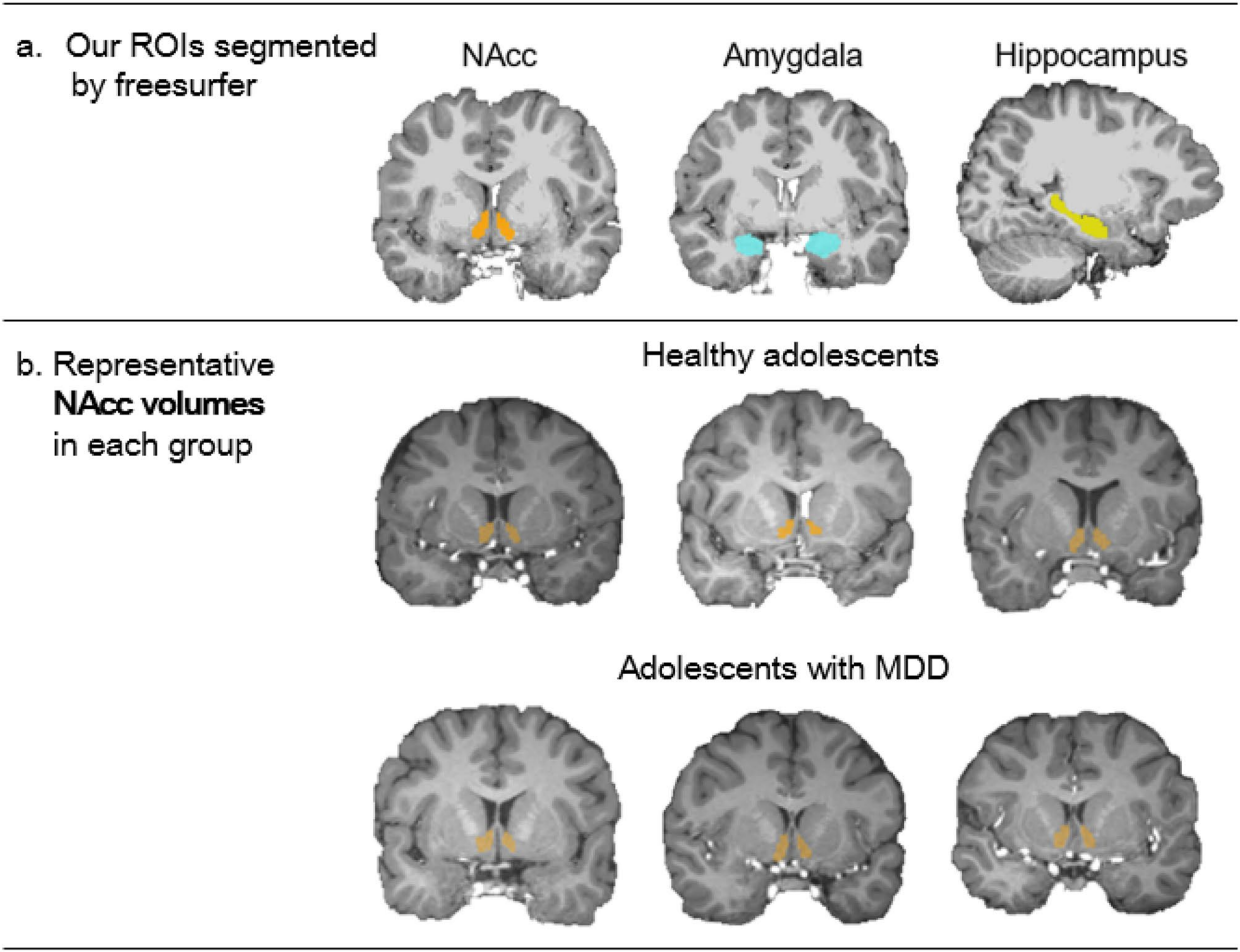
(**a**) Examples of our regions of interest (ROIs) segmented by Freesurfer, (**b**) Examples of representative nucleus accumbens (NAcc) volumetric images in each group.

There was a significant group difference in age, but not in any other demographic variables. MDD adolescents were older than healthy controls. Adolescents with MDD showed more severe depressive and anxiety symptoms and reported higher peer problems compared to healthy controls (see Figure S1a–c, for individual values). As we mentioned above, we focused on volumetric alteration in three predetermined subcortical regions including the amygdala, hippocampus, and NAcc. Figure 1a shows examples of segmented these regions. There was a significant group difference in the NAcc volume, but not in the amygdala and hippocampal volumes. MDD adolescents showed larger NAcc volume (7.59%) compared to healthy controls (Figure S1d). A few examples of segmented NAcc volumes in each group are also presented in Fig. 1b. Effect sizes of significant results ranged from medium (Cohen’s *d* = 0.41 and Partial *η*^2^= 0.08–0.17) to large (Partial *η*^2^= 0.45–0.75) (Table 1).

### Correlation analysis

Partial correlation coefficients were calculated to examine relationships between continuous variables (peer problems, brain volumes, and depressive symptom severity), controlling for age, gender, IQ and intracranial volume (ICV). Peer problems were positively correlated with depressive symptoms (*r* = 0.53, *p* < 0.001, Cohen’s *f*^2^ = 0.39). Peer problems were also significantly correlated with NAcc volume (*r* = 0.22, *p* < 0.05, Cohen’s *f*^2^ = 0.05), but not with amygdala volume (*r*  = − 0.13, *p* = 0.14) and hippocampal volume (*r* = 0.09, *p* = 0.32). NAcc volume was significantly correlated with depressive symptoms (*r* = 0.27, *p* < 0.01, Cohen’s *f*^2^ = 0.08), but amygdala and hippocampal volumes were not significantly correlated with depressive symptoms (*r* = − 0.09, *p* = 0.34 and *r* = 0.08, *p* = 0.37, respectively). Effect sizes ranged from small (Cohen’s *f*^2^ = 0.05–0.08) to large (Cohen’s *f*^2^ = 0.39). The scatter plots of significant correlations between variables are presented in Figure S2 (see supplementary materials).

### Indirect effects of peer problems on adolescent depression (MDD vs. CON)

**Figure 2:**
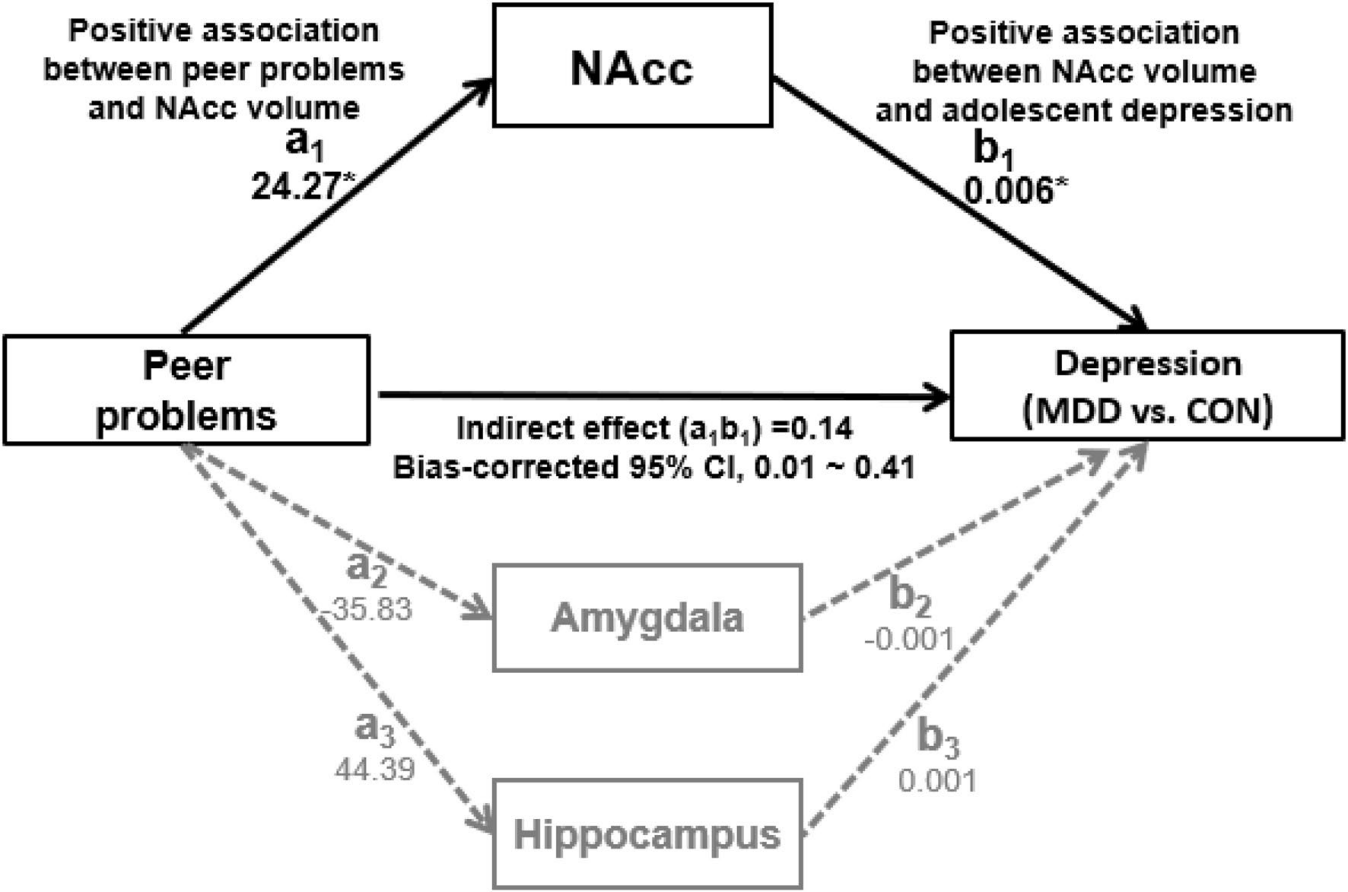
The model describing the associations between peer problems, subcortical volumes, and adolescent depression, controlling for age, gender, IQ, and intracranial volume (ICV). NAcc = Nucleus accumbens, MDD = Major Depressive Disorder, CON = healthy controls, a_1_ = unstandardized regression coefficient indicating the effect of peer problems on NAcc volume, b_1_ = unstandardized regression coefficient indicating the effect of NAcc volume on adolescent depression (MDD vs. CON), a_2_ = unstandardized regression coefficient indicating the effect of peer problems on amygdala volume, b_2_ = unstandardized regression coefficient indicating the effect of amygdala volume on adolescent depression (MDD vs. CON), a_3_ = unstandardized regression coefficient indicating the effect of peer problems on hippocampus volume, b_3_ = unstandardized regression coefficient indicating the effect of hippocampus volume on adolescent depression (MDD vs. CON), *p < .05.

The results of the indirect effects of peer problems on adolescent depression via three subcortical volumes are presented in Table 2 and Fig. 2. As shown in Table 2 and Fig. 2, the effect of peer problems on NAcc volume (a_1_) and effect of NAcc volume on MDD (b_1_) were significant. Our analysis revealed a significant indirect effect of peer problems on adolescent depression (MDD vs. CON) through the NAcc volume, 0.14, 95% Bootstrap CI (0.01, 0.41), but not through the amygdala volume, 0.03, 95% Bootstrap CI (− 0.06, 0.20) and the hippocampus volume, 0.03, 95% Bootstrap CI (− 0.05, 0.17). This result indicated that the significant indirect effect through enlarged NAcc volume emerged between peer problems and adolescent depression. There was one participant who was taking ADHD medication. We conducted the same analysis without this participant, and confirmed that our findings remained significant.

**Table 2:** A summary of multiple mediation analysis for peer problems, subcortical volumes, and adolescent depression.

Independent variable (IV)	Multiple mediators (M)	Dependent variable (DV)	Effect of IV on M	Effect of M on DV	Direct effect	Indirect effect	95% CI	
			a_i_	b_i_	cʹ	(a_i_ × b_i_)	LL	UL
		**Categorical DV**^j^						
Peer problems	1. NAcc	Group (MDD vs. CON)	24.27 (SE = 10.13)*	0.006 (SE = 0.002)*	1.25 (SE = 0.33)***	0.14 (SE = 0.10)*	0.008	0.410
	2. Amygdala		− 35.83 (SE = 24.30)	− 0.001 (SE = 0.001)		0.03 (SE = 0.06)	− 0.060	0.199
	3. Hippocampus		44.39 (SE = 44.69)	0.001 (SE = 0.001)		0.03 (SE = 0.05)	-0.052	0.169
		**Continuous DV**^k^						
Peer problems	1. NAcc	Depressive symptoms	24.27 (SE = 10.13)*	0.029 (SE = 0.013)*	8.71(SE = 0.33)***	0.70 (SE = 0.44)*	0.022	1.742
	2. Amygdala		− 35.83 (SE = 24.30)	− 0.007 (SE = 0.006)		0.23 (SE = 0.33)	− 0.260	1.067
	3. Hippocampus		44.39 (SE = 44.69)	0.002 (SE = 0.003)		0.10 (SE = 0.23)	− 0.233	0.729

^j^Categorical dependent variable (DV) diagnosed by the Kiddie-Schedule for Affective Disorders and Schizophrenia for School-Age Children-Present and Lifetime (K-SADS-PL) Version.

^k^Continuous dependent variable (DV), depressive symptom severity assessed by the Children’s Depression Rating Scale-Revised (CDRS-R), SE = standard error, CI = confidence interval, LL = lower limit, UL = upper limit, *p < .05, ***p < .001.

### Sensitivity analysis

#### Indirect effect of adolescent depression (MDD vs. CON) on peer problems

Our cross-sectional design limited our ability to interpret our finding regarding causal relationships. Despite such limitation, we attempted to show our proposed model that best described the relationships among variables by investigating the indirect effects of adolescent depression on peer problems via subcortical volumetric alteration. However, there were no significant indirect effects of adolescent depression (MDD vs. CON) on peer problems through subcortical volumes including the NAcc volume, 0.09, 95% Bootstrap CI (− 0.02, 0.24), the amygdala volume, 0.02, 95% Bootstrap CI (− 0.07, 0.13) and the hippocampal volume, 0.03, 95% Bootstrap CI (− 0.03, 0.13).

#### Indirect effects of peer problems on depressive symptom severity (continuous variable assessed by CDRS-R)

We also tried to confirm our main finding using continuous depressive symptoms as a dependent variable. This analysis revealed a similar result (Table 2). There was a significant indirect effect of peer problems on depressive symptoms through the NAcc volume, 0.70, 95% Bootstrap CI (0.02, 1.74), but not through the amygdala volume, 0.23, 95% Bootstrap CI (− 0.26, 1.07) and the hippocampus volume, 0.10, 95% Bootstrap CI (− 0.23, 0.73).

#### Indirect effects of peer problems on anxiety symptoms

Additional analyses were conducted to test whether our model was specific to depression, or was generalizable to internalizing problems of depression and anxiety. Peer problems were significantly correlated with anxiety symptoms (*r* = 0.54, *p* < 0.001, Cohen’s *f*^2^ = 0.41). NAcc volume was also significantly correlated with anxiety symptoms (*r* = 0.25, *p* < 0.01, Cohen’s *f*^2^ = 0.06), but amygdala and hippocampal volumes were not significantly correlated with anxiety symptoms (*r* = − 0.09, *p* = 0.34 and *r* = − 0.00, *p* = 0.97, respectively). There were no significant indirect effects of peer problems on anxiety symptoms through subcortical volumes such as the NAcc volume, 0.61, 95% Bootstrap CI (− 0.03, 1.53), the amygdala volume, 0.08, 95% Bootstrap CI (− 0.45, 0.72), and the hippocampal volume, − 0.09, 95% Bootstrap CI (− 0.42, 0.35).

## Discussion

This study examined the role of subcortical brain alteration in linking peer problems with depression in adolescence to better understand the relationship between these three variables. We hypothesized that peer problems would be associated with adolescent depression through volumetric alteration in subcortical regions including the amygdala, hippocampus, and NAcc. Consistent with our hypothesis, there was a significant indirect effect of peer problems on adolescent depression via increased NAcc volume. This finding indicates that NAcc volume may be one possible structural alteration that plays an important role in linking peer problems as social stress with depression in adolescence. However, the indirect effects of peer problems on adolescent depression via the amygdala and hippocampal volumes were not significant.

Notably, peer problems had the indirect effect on adolescent depression through only NAcc volumetric alteration. This result may be in line with the animal models of depression, which proposed that social stress induced depressive-like behaviors through altered neurobiological factors such as molecular, cellular and morphologic changes in the NAcc. While the NAcc is a very-well known region involved in reward and reward-based learning^30^, it has also been implicated in aversion, punishment, and pain processing^31^. It is important to note that the NAcc shows dramatic changes and plays a significant role in stress-related processing and development of depression during adolescence^10,32^. Increased NAcc volume may reflect heightened sensitivity to social threat or pain, which is acquired from adverse peer relationships. In other words, repeated or constant involvement in bullying may heighten the adolescents’ sensitivity to social evaluation and increase social stress contributing to NAcc alteration. It has also been theorized that such heightened sensitivity to social evaluative threat may be an important vulnerability factor for adolescent depression^33^. Thus, larger NAcc volume as an indicator of heightened social stress from peer relational problems may play an important role in the onset and maintenance of adolescent depression.

Inconsistent with our hypothesis, there were no significant indirect effects of peer problems on adolescent depression via amygdala and hippocampal volumetric alteration. A few reasons could explain this discrepancy. First, the types of social contexts and social stress may matter because altered volumes in the amygdala and hippocampus were often associated with early adverse life events such as maltreatment^34,35^, but not with problematic peer relationships in human studies^36^. The amygdala and hippocampus have been known to play important roles for early emotional learning (e.g., appropriate emotional reactivity and regulation) acquired from family contexts. Another reason may be related to the different time frames on when one is exposed to social stress, and when the volumetric changes become visible. For example, hippocampal volumes were more significantly affected by early separation stress (childhood stress) compared to adolescent stress^34^. Cross-sectional studies often showed that early maltreatment was associated with adult hippocampal volume changes, but not with childhood volume changes, suggesting that there exists a temporal delay in showing changes in volume^13^. Thus, it is possible that the amygdala and hippocampal volume alterations may be visible during adulthood or years after peer problems affect the structural changes during adolescence. Future longitudinal research that assesses early family stress and peer problems during adolescence, and follows up with structural changes is needed to better understand the volumetric alterations in the amygdala and hippocampus associated with early family stress and peer problems.

It is also important to note that the indirect effects of adolescent depression on peer problems through subcortical volumetric alteration were not significant. Such null finding from the reverse model linking depression to peer problems via subcortical alteration may provide additional support for our proposed model. Possibly, our participants experience peer problems that may contribute to NAcc alteration prior to having depression. Indeed, about 77% of our MDD adolescents had a first-episode of MDD when they were recruited for this study, suggesting that they may be more likely to have past or continued peer problems. However, caution is needed in this interpretation due to our cross-sectional design and future longitudinal study is required to confirm this finding.

Unlike the association between peer problems and adolescent depression (and depressive symptoms), there were no significant indirect effects of peer problems on anxiety symptoms via subcortical volumetric alteration. As mentioned above, the NAcc is involved in threat or avoidance, which are both core features of anxiety^37^. Previous adult studies have shown that larger NAcc volume was correlated with trait anxiety^38^. These results provided some evidence of the association between NAcc volume and anxiety, but these results are relatively unclear in the case of adolescence. A recent longitudinal adolescent study has demonstrated that volumetric changes in the putamen and caudate mediated the association between peer victimization and anxiety^36^. This result may indicate that volumetric alteration in the different striatal regions (e.g., dorsal striatum such as caudate vs. anterior striatum such as NAcc) plays a significant role in linking peer problems and anxiety. However, future longitudinal research is needed to investigate specific or general roles of NAcc volumetric changes in linking peer problems and affective disorders (e.g., depression and anxiety) in adolescence.

This study had some imitations. First, we used the cross-sectional design, which limited our ability to understand the causal relationships between peer problems, NAcc volumetric alteration, and adolescent depression. Future longitudinal MRI studies using both animal and human subjects are needed to examine the causal relationships between the variables and to better understand roles of cellular and structural brain alteration in linking peer problems and adolescent depression. Second, we measured peer problems using self-report measures, and thus scores may be biased. In addition, we did not collect information regarding the duration and frequency of peer problems (i.e., bulling involvement), and whether peer problems indicate current or past problems with peers, and so on. Such limited information regarding peer problems may restrict us on comprehensively interpreting our findings. For example, given that the timing and chronicity of social stress matter^35^, it is difficult to understand some trajectory of NAcc volumetric alteration when experiencing peer relational problems. Third, we only used the brain structure data (i.e., subcortical volumes), so future research that examines whether functional alternations (e.g., altered neural responses to peer rejection) in the NAcc play a similar role in linking peer problems and adolescent depression is needed. Fourth, although age-matched MDD and healthy adolescents were initially recruited, there was a significant difference in age between MDD adolescents and healthy controls included in our final sample. Age was significantly correlated with depressive symptoms assessed by the CDRS-R, but not with any other variables including peer problems and subcortical volumes. We controlled for potential age effects on our findings by including age as a covariate into the analyses. Fifth, peer problems were assessed by a sum of peer victimization and bullying behavior scores. It is possible that adolescents who had higher peer problems in our study could have higher scores of only peer victimization (‘victims’ group), higher scores of only bullying behaviors (‘bullies’ group), or higher sum of both measures (‘bully/victims’ group). However, small sample sizes of each group limits our ability to test our mediation model separately. Future research may be needed to test whether our mediation model is supported in pre-selected groups of ‘victims’, ‘bullies’, and ‘bully/victims’ to clarify the specificity and commonality for different groups of peer problems. Finally, we did not survey other sources of adversity, such as maltreatment or exposure to domestic violence, that might also have an impact on subcortical structure, and on adolescent depression.

These limitations notwithstanding, our main finding was the unique role of NAcc volumetric alteration in linking peer problems to depression, but not linking depression to peer problems in adolescence. This finding highlights that NAcc volumetric alteration may be one of critical neurobiological factors for linking peer problems and adolescents. To our knowledge, this study is one of the first research to provide evidence that peer problems have indirect effects through NAcc volumetric alteration on adolescent depression. Overall, our finding suggests that altered NAcc volume may serve as a pathway through which peer problems may contribute to adolescent depression.

## Methods

### Participants

Healthy control adolescents and adolescents with major depressive disorder (MDD) in the age group of 12–17 years were recruited from the Seoul National University Hospital in Korea. A total of 152 adolescents, including 95 with MDD and 57 healthy controls, were initially recruited for this study. Five participants withdrew their consents after screening and one participant dropped out before the MRI assessment. Of the remaining participants, twenty-one subjects were excluded due to incidental findings (e.g., arachnoid cyst) (N = 5), artifact such as motion and noise (N = 15), and incomplete self-report data (N = 1). Our final sample was comprised of 78 adolescents with MDD (age mean [SD] = 14.9 ± 1.5, 56 girls) and 47 healthy controls [14.3 ± 1.4, 26 girls]). MDD adolescents were diagnosed based on DSM-5^39^ criteria using the Kiddie-Schedule for Affective Disorders and Schizophrenia for School-Age Children-Present and Lifetime Version (K-SADS-PL)^40,41^. Thus, MDD adolescents were included if they had a current diagnosis of MDD according to DSM-5 criteria using the K-SADS-PL. MDD adolescents were excluded if they had (a) any chronic medical diseases, (b) a history of psychotic disorders including schizophrenia or bipolar disorder, (c) a history of eating disorder, (d) any developmental disorders such as autism, (e) a history of alcohol or other substances abuse within the past 6 months, (f) any neurological or physical diseases, (g) first degree relatives with a history of bipolar I disorder, (h) any psychiatric medications (except treatments for ADHD). Healthy controls did not have any history of psychiatric illness and were excluded if they had first degree relatives with a history of any psychiatric disorders. Furthermore, all participants were excluded if their intelligence quotient (IQ) was below 70.

### Procedure

This study was approved by the institutional review board for human subjects at the Seoul National University Hospital. The parents provided informed consent and adolescents provided assent using forms approved by Seoul National University Hospital Review Board. All methods were carried out in accordance with relevant guidelines and regulations. Participants completed the interview and questionnaires assessing depressive, anxiety symptoms and peer problems. Afterwards, participants were given MRI sessions to collect their structural T1 images.

### Clinical assessments and self-report measures

The participants completed the Children’s Depression Rating Scale-Revised (CDRS-R)^42^ and the Peer-Victimization Scale (PVS) & Bullying-Behavior Scale (BBS)^43^, which assessed the depressive symptom severity and peer problems. Although our participants were primarily categorized as healthy controls and MDD adolescents based on the K-SADS-PL, they were also assessed for their depressive symptom severity using the CDRS-R. The CDRS-R is widely used for the assessment of depression severity in children and adolescents. It includes 17 symptoms-related items, derived from the adult Hamilton Depression Rating Scale, which are rated by clinical interviewers based on the summary of child and parent reports and their behavioral observation during the interview. Excellent internal consistency of the Korean version of the CDRS-R was reported (Cronbach’s α = 0.91)^44^. This scale had good internal consistency in this sample (Cronbach’s α = 0.93).

Two self-report scales, PVS and BBS were used to measure peer victimization and bullying behaviors, respectively. The PVS includes 6 items regarding being a victim of negative physical (e.g., being hit and pushed) and verbal behaviors (e.g., being teased and laughed at). Each item consists of two opposite statements (e.g., “Some kids are often teased by other kids BUT other kids are not teased by other kids”). The BBS contains 6 items regarding physical and verbal bullying behaviors (e.g., teasing). Similar to PVS, each item includes two opposite statements (e.g., “Some kids often tease other kids BUT other kids do not tease other kids”). Participants were asked to choose which statement better represents them, and then to rate the statement as “really true for them” or “sort of true for them” on six PVS and six BBS items. Responses were scored on a scale of 1–4 (two statements × two scales for each item), with higher scores indicating higher levels of peer problems (i.e., bullying involvement as either victims or bullies). The validated Korean versions of the PVS and BBS were used^45^ in this study. These measures demonstrated strong internal consistencies (PVS: Cronbach’s α = 0.77 and BBS: Cronbach’s α = 0.75)^45^. These scales had good internal consistency (PVS: Cronbach’s α = 0.62, BBS: Cronbach’s α = 0.83 and combined Cronbach’s α = 0.83) in the current sample. Given our interest in the indirect effects of peer problems as social stress on depression via subcortical volumetric alteration, we computed the total scores of PVS and BBS and used those total scores as a proxy of “peer problems” in this study. Peer relational problems (i.e., involvement in bullying) were regarded as major stressful life events for adolescents who bullied or were victimized^46^. Furthermore, peer problems have been suggested as possible common characteristics of bullies and victims of bullying^47,48^.

Additionally, the Screen for Child Anxiety Related Emotional Disorder (SCARED)^49^ was used to assess anxiety symptoms. The SCARED is a child- and parent-report questionnaire with 41 items (e.g., I feel nervous with people I don’t know well) assessing symptoms of anxiety disorders. We used scores of the child-report SCARED. Each item is answered on a 3-point scale (0 = almost never, 1 = sometimes, 2 = often). The Korean version showed good internal consistency (Cronbach’s α range = 0.60–0.86)^50,51^. Internal consistency was excellent in the current sample (Cronbach’s α = 0.97).

### MRI acquisition and T1 image processing

High-resolution structural T1 images were collected using a Siemens 3 T MR scanner (Trio Tim; Siemens, Erlangen, Germany) with a 12-channel birdcage head coil. A T1-weighted 3D gradient echo pulse sequence with magnetization-prepared rapid gradient-echo sequencing were used to obtain the T1 image (TR = 1900 ms, TE = 3.13 ms, flip angle = 9°, slice thickness = 0.9 mm, matrix size = 256 × 224 × 176). We visually inspected T1 images and then excluded data with excessive head motion and incidental findings (e.g., arachnoid cyst). The structural T1 image was first processed using the Freesurfer 6.0 package (https://surfer.nmr.mgh.harvard.edu/). We used the default processing pipeline, called “recon-all’ (https://surfer.nmr.mgh.harvard.edu/fswiki/recon-all/). This pipeline includes motion correction, intensity normalization, Talairach transformation, and skull stripping. Details for these steps of processing have been well described in a previous study^52^. This also allowed us to do automatic segmentation on the subcortical regions including amygdala, hippocampus and NAcc (Fig. 1a), and to calculate the ICV.

We inspected the processed T1 images and found erroneous white matter segmentation near the parietal cortex areas. To correct this problem, we used the “control points” (https://surfer.nmr.mgh.harvard.edu/fswiki/FsTutorial/ControlPoints_freeview/) and re-ran part of the “recon-all”. Afterwards, we confirmed whether the white matter segmentation was corrected.

### Statistical analysis

Demographic characteristics were analyzed using independent-sample *t*-tests for age and intelligence (IQ) and a Chi-square test for gender. Clinical variables were compared between CON and MDD using one-way analysis for covariance while controlling for age, gender, and IQ. Subcortical volumes were compared between CON and MDD using one-way analysis for covariance while controlling for age, gender, IQ, and ICV. The PROCESS macro for SPSS version 25.0^53^ was conducted to examine the indirect effects of peer problems on adolescent depression (MDD vs. CON), controlling for age, gender, IQ, and ICV. Three subcortical volumes including the NAcc, amygdala, and hippocampus were entered as mediators into the model simultaneously (Fig. 2). This analysis was performed using bootstrapping (i.e., 95% bias-corrected bootstrap confidence intervals [CI] for the indirect effects based on 10,000 bootstrap resamples). Indirect effects were considered significant if the 95% bias-corrected CI did not include zero^54^.

## Supplementary information

Supplementary Information 1.
