# Supplementary material for: The indirect effect of peer problems on adolescent depression through nucleus accumbens volume alteration
Source: Sci Rep. 2020 Jul 30;10:12870. doi: 10.1038/s41598-020-69769-3 (PMC7392894; doi:10.1038/s41598-020-69769-3)
Supplement: Supplementary file 1 — Supplementary Information 1. [file 41598_2020_69769_MOESM1_ESM.docx]

**Supplementary materials**

**The indirect effect of peer problems on adolescent depression through**

**nucleus accumbens volume alteration**

Kyung Hwa Lee, Jae Hyun Yoo, Jung Lee, Seong Hae Kim, Ji Youn Han, Soon-Beom Hong, Jiyoon Shin, Soo-Churl Cho, Jae-Won Kim, David A. Brent


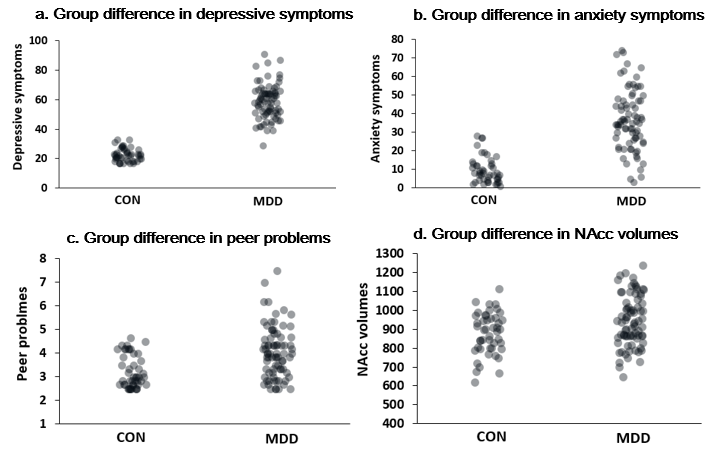


**Figure S1.** Grouped column scatter plots showing individual values in each group

Note. NAcc = Nucleus accumbens, CON = healthy controls, MDD = Major Depressive Disorder


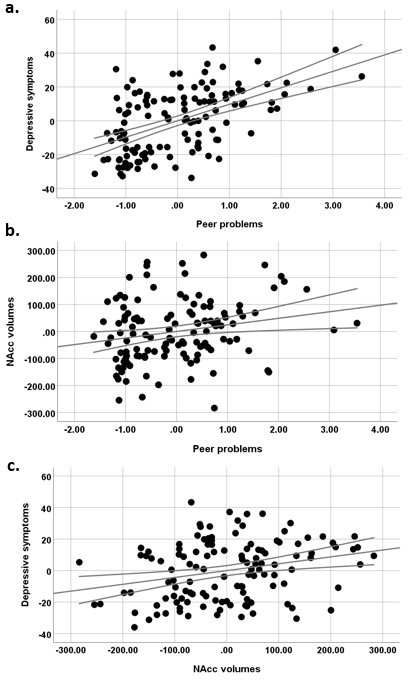


**Figure S2.** a) Partial correlation scatter plot showing a positive correlation between peer problems and depressive symptoms (*r* = .53, *p* <.001, *f^2^* = 0.39), b) Partial correlation scatter plot showing a positive correlation between peep problems and NAcc volumes (*r* = .22, *p* < .05, *f^2^* = 0.05), and c) Partial correlation scatter plot showing a positive correlation between NAcc volumes and depressive symptoms (*r* = .27, *p* < .01, *f^2^* = 0.08). All partial correlation coefficients were computed after controlling for age, gender, IQ, and ICV.

Note. NAcc = Nucleus accumbens, ICV = Intracranial volume
